# *Moringa oleifera* Extracts as Strategic Phyto‐Therapy for Alzheimer's Disease

**DOI:** 10.1002/fsn3.70007

**Published:** 2025-03-26

**Authors:** Rita Carrotta, Silvia Vilasi, Maria Assunta Costa, Fabio Librizzi, Vincenzo Martorana, Rosa Passantino, Carla Buzzanca, Vita di Stefano, Maria Grazia Ortore, Silvia Piccirillo, Alessandra Preziuso, Simona Magi, Maria Rosalia Mangione

**Affiliations:** ^1^ Institute of Biophysics, National Research Council Palermo Italy; ^2^ Department of Science and Biological, Chemical and Pharmaceutical Technologies STEBICEF University of Palermo Palermo Italy; ^3^ Department of Life and Environmental Sciences Marche Polytechnic University Ancona Italy; ^4^ Department of Biomedical Sciences and Public Health, School of Medicine Marche Polytechnic University Ancona Italy

**Keywords:** Alzheimer's disease, amyloid inhibition, functional foods, neuroprotection, polyphenols

## Abstract

In the last decades, plant extracts have received great attention. In particular, many studies pointed out the potential neuroprotective effect of polyphenols‐rich extracts from plants. Evidence indeed highlights the action of polyphenols, both as antioxidants and as inhibitors in the formation of amyloid protein aggregates, known to be involved in neurodegenerative diseases. In this work, aqueous extracts obtained at high and room temperature from leaves of *Moringa oleifera* (MO) harvested in Sicily were characterized for polyphenolic content, anti‐oxidative and free radical scavenging capacity. UHPLC‐HESI‐MS analysis shows that both water extracts are rich in terms of polyphenols. Then, MO aqueous extracts were tested as inhibitors in the amyloid aggregation process of Amyloid β‐peptide 1–42 (Aβ_1‐42_). This peptide is strongly involved in Alzheimer's disease (AD), its overproduction and aggregated species being considered a hallmark of AD. Results show that MO extracts cause a strong inhibition on the amyloid process. Biophysical characterization of the extracts reveals the presence of stable polyphenol assemblies. Both free and aggregated polyphenols elicit an efficient inhibition mechanism held up by their ability to interact with metastable species, strongly hindering autocatalytic amyloid growth. Finally, the effects of the MO room temperature extract have been tested on an AD cell model, retinoic acid‐differentiated SH‐SY5Y cells challenged with glyceraldehyde (GA). Cell pretreatment with MO extract results in an improved cell viability in comparison with the control and furthermore in the reduction of both mitochondrial reactive oxygen species and GA‐stimulated Aβ_1‐42_ production.

## Graphical abstract

*Moringa oleifera* green extraction with water leads to a high polyphenols yield. Both, high and room temperature extracts, inhibit amyloid beta‐peptide fibril formation. Room temperature extract protects from typical Alzheimer's disease cell alterations.

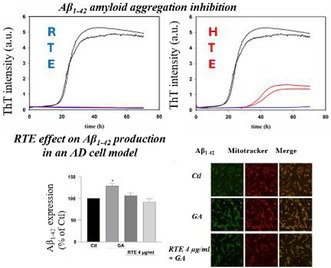

## 1 Introduction

In the last decades the habit of a proper and balanced food consumption and a good lifestyle have been correlated to a better aging condition (Leitão et al. 2022; Tittikpina et al. 2019). In this context, research on food and its components has been a main scientific issue. This interest has been especially directed in understanding how natural molecules can play a role in physiological mechanisms, and how to use this knowledge for developing new therapeutic strategies. In fact, the more life expectancy increases, the more the prevalence of chronic diseases such as diabetes, cardiovascular diseases and neurodegenerative diseases increases. Thus, the interest in molecules which can modulate or be implied on healthy biochemical pathways has surged. The discovery of potential benefits from natural molecules, which are constitutively present in food or can be included as supplements in a diet, is a strategic way to prevent the onset or calm down symptoms of age‐related pathologies (Ali et al. 2020; Li et al. 2023; Maccioni et al. 2022). Among natural molecules, polyphenols, compounds richly present in fruits, vegetables and cereals, have received great attention due to their properties. Several authors have shown the potential of polyphenols in counteracting cellular oxidative and inflammatory processes which are the basis of several pathological mechanisms (cancer, diabetes, cardiovascular, and neurodegenerative diseases) (Ali et al. 2020; Rana et al. 2022; Rudrapal et al. 2022). Moreover, this class of molecules can interfere with protein amyloid aggregation, a process common to many neurodegenerative diseases (including Alzheimer's disease, Parkinson's disease, Huntington's disease, Amyotrophic Lateral Sclerosis, and prion disease) (Borana et al. 2014; Boubakri et al. 2020; Glabe 2006; Hanaki et al. 2016; Tinku and Choudhary 2023). Alzheimer's disease (AD) represents the most common form of dementia in the elderly and is characterized by the extracellular aggregation and accumulation of Amyloid β‐peptide (Aβ) in well‐ordered β‐sheet rich fibers. Fibrils deposition, the hallmark of AD, represents indeed the final step of a pathway involving protein misfolding and aggregation passing through the formation of oligomers (small spherical aggregates and protofibrils) which seem to be the causative primary toxic species (Haass and Selkoe 1993, 2007; Kayed and Lasagna‐Reeves 2013; Mangione et al. 2015; Pike et al. 1993). These species interact with cell membranes causing alteration of functionality (Matsuzaki 2014; Ricci et al. 2019).

*Moringa oleifera* (MO), a fast‐growing tree originating from the sub‐Himalayan area, widely present in eastern, western and central Africa, Arabia, south‐eastern Asia, the Pacific, the Caribbean, and South America, has been attracting much interest in the last years for its therapeutic potentiality (Agbogidi and Ilondu 2012). This plant has been successfully used in traditional medicine in many ancient civilizations to treat several diseases and infections, so capitalizing its anti‐inflammatory, antimicrobial, antioxidant, and anticancer properties (Arora and Arora 2021; Azlan et al. 2023; Fahey 2005; Farooq et al. 2012; García‐Beltrán et al. 2020; Ghimire et al. 2021; González‐Romero, Guerra‐Hernández, and Rodríguez‐Pérez 2022; Li et al. 2023; Omotoso et al. 2015; Stohs and Hartman 2015). Besides being a good source of protein, vitamin, oil, fatty acid, micro–macro mineral elements, it has been used to improve nutrition and boost food security and it has received attention as a fodder in animal husbandry (Fahey 2005; Mahfuz and Piao 2019; Nuzzo et al. 2021; Peñalver et al. 2022; Su and Chen 2020). Moreover, every part of MO plant, including leaves, bark, seeds, flowers and pods, is edible, and rich of compounds important for human and livestock wellness, adding sustainability value to its use and cultivation. These are some of the reasons why MO, also called the Tree of Miracles, is arousing so much interest in the scientific community.

In this context, our aim is to investigate whether the bioactive compounds from a MO plant grown in Sicily (Cirlini et al. 2022) are able to counteract the aggregation of the Aβ peptide and/or its toxic effects.

Furthermore, we studied different extraction methods from dry MO leaves. Specifically, we explored water‐based extraction protocols at both room temperature and boiling temperature. The use of water as a solvent for extraction is advantageous because it is safe, inexpensive, and environmentally friendly. To evaluate the efficiency of water‐based extractions, comparative extraction with 50% ethanol/water was also performed. The determination of total polyphenols content was obtained by Folin–Ciocâlteu method, the anti‐oxidative properties of the extracts were investigated with Oxygen Radical Absorbance Capacity (ORAC), the composition of polyphenols was investigated with mass spectrometry detection (UHPLC‐MS), and the extracts structural characteristics were studied by Atomic Force Microscopy (AFM) and Light Scattering (LS) measurements. The direct effect of MO water extracts on the Aβ peptide aggregation was monitored by Thioflavin T fluorescence assay (ThT), Far‐UV Circular Dichroism (CD) and AFM. Cell viability and the protective effect of MO water extracts against oxidative stress were studied on the LAN5 neuroblastoma human cell line. Lastly, the protective role of the MO room temperature water extract versus the typical cell alterations observed in AD was evaluated on retinoic acid‐differentiated SH‐SY5Y cells challenged with glyceraldehyde (GA).

## 2 Materials and Methods

### 2.1 Preparation of *Moringa Oleifera* (MO) Extracts

Dry MO leaves, kindly supplied from a local grower, were crushed in a mortar with liquid nitrogen to obtain a fine powder. For the extraction at room temperature 0.3 g of the powder was mixed with 20 mL of water or with 20 mL of an ethanol‐water mixture (50:50 v/v). Samples were sonicated for 20 min at 20°C and then incubated in the dark at 20°C for 3 days. For the high temperature extraction 0.3 g of powder was added to 20 mL of boiling water for 5 min. At the end of the extraction procedure, all samples were filtered with a gauze to eliminate the coarse particles and then subsequently filtered with 0.8 μm and 0.2 μm filters (sartorius CA) and finally aliquoted and stored at −80°C.

### 2.2 Chemicals and Reagents

Methanol, ethanol, acetonitrile, sodium carbonate, sodium bicarbonate, gallic acid, sodium chloride, and Folin–Ciocâlteu reagent, Thioflavin T, 2,2'‐azobis(2‐methylpropionamidine) dihydrochloride (AAPH), Ethyl myristate, quercetin, rutin, kaempferol, apigenin, chlorogenic acid, Trolox and 2,2'‐azobis(2‐methylpropionamidine) dihydrochloride were purchased from Sigma‐Aldrich (St. Louis, MO, USA). The synthetic Aβ_1‐42_ peptide was purchased from Anaspec. HPLC‐grade water was obtained by purifying double distilled water in a Milli‐Q Gradient A10 system (Millipore, Bedford, MA, USA).

### 2.3 Total Phenol Content (TPC) Determination

TPC of all the extracts was determined using the Folin–Ciocâlteu assay in agreement with Gutfinger (Gutfinger 1981) with slight modifications. Briefly, 20 μL of extract were mixed with 500 μL of distilled water and 50 μL of Folin–Ciocâlteu's reagent. After 3 min 100 μL of Na_2_CO_3_ and 350 μL of water were added. The mixture was incubated for 90 min at room temperature in the dark. The absorbance was measured at 765 nm wavelength against a reagent blank using a Shimadzu (2401 PC UV) spectrophotometer. Gallic acid was used as a standard for preparing the calibration curve ranging 50–500 mg/L. The TPC was expressed as mg of gallic acid equivalent (GAE) per gram of dry leaves. All assays were carried out in triplicate.

### 2.4 Oxygen Radical Absorbance Capacity (ORAC) Assay

The ORAC assay was performed according to Ninfali et al. (Ninfali et al. 2002) with slight modifications. In brief, the reaction mixture was prepared in a 96‐well black microplate as follows: 160 μL of 0.04 μM Fluorescein in 75 mM NaK phosphate buffer pH 7.0, 20 μL of appropriately diluted sample or 20 μL of 100 μM Trolox used as reference standard. Each mixture was kept 10 min at 37°C in the dark, and the reaction was started by adding 20 μL of 40 mM AAPH. The fluorescence decay was measured at 37°C every 2 min at 485 nm excitation and 538 nm emission wavelengths respectively, using a Fluoroskan Ascent F2 Microplate (Thermo Fisher Scientific, Massachusetts, USA). The ORAC value for each sample was expressed by μmoles of Trolox Equivalents (μmol TE) per gram of MO dry leaves, with the following equation:

(1) $$\text{ORAC value}\;\left(\text{μmol}\;\mathrm{TE}g^{-1}\right)=k∙a∙h\;\left[\frac{\left(S_{\text{sample}}-S_{\text{blank}}\right)}{\left(S_{\text{Trolox}}-S_{\text{blank}}\right)}\right]$$

where *k* is the total dilution of the extract; *a* is the ratio between the volume (liters) of the extract and grams of dried plants; *h* is the final concentration of Trolox expressed as μmol/L and S is the area under the curve of fluorescein emission vs. time, in the presence of (i) sample, (ii) Trolox, or (iii) extraction buffer (blank).

### 2.5 Polyphenols Extraction and UHPLC‐HESI‐MS Analyses

For the qualitative‐quantitative analyses of phenolic compounds, each extract was filtered through a 0.22 μm PTFE syringe filter (Whatman) into glass vials before UHPLC‐HESI‐MS analysis. For quantitative determination of polyphenols, five calibration curves (quercetin, rutin, apigenin, chlorogenic acid, and kaempferol) in a range of concentration of 2.5–25 ppm, were developed. The identification and quantification of polyphenols were performed by UHPLC‐HESI‐MS analysis using Q‐Exactive LCq/Orbitrap MS (ThermoFisher Scientific), interfaced with UHPLC Ultimate 3000 RS (Dionex), equipped with HESI (Heated Electrospray Ionization) source and a Luna column C18(2) (150 × 2.0 mm, 5 μm stationary phase), provided by a pre‐column. The column was maintained at 30°C and the flow rate was 400 μL /min. The mobile phase solutions were H_2_O with 0.1% formic acid (A) and methanol with 0.1% formic acid (B). The binary gradient separation was as follows: 0–2 min 95% A; 2–18 min 95%–5% A; 18–20 min 5% A; 20–40 min 5%–95% A. The initial conditions were maintained for 3 min to equilibrate the column. 1 μL of each sample was injected with a run time of 40 min. The HESI source conditions were set as follows: spray voltage (±): 3000.00; capillary temperature (±): 300.00; sheath gas (±): 30.00; aux gas (±): 15.00; S‐Lens RF level: 50.00. Data were collected in both positive and negative ionization modes. The scan range was set between 80 and 1000 m/z.

### 2.6 Preparation of Aβ_1‐42_—MO Samples

The synthetic Aβ_1–42_ peptide was solubilized in 5 mM NaOH (Sigma‐Aldrich), pH 10, and lyophilized according to Fezoui et al. (Fezoui et al. 2000). The lyophilized peptide was then dissolved in 50 mM sodium phosphate buffer with 20 mM NaCl at pH 7.4. Samples were filtered using a 0.2 μm pore size syringe filter (Millex—LG) and subsequently a supplementary filtration was realized by 30 KDa cut‐off centrifuge filtration (Amicon Ultra 4 Millipore). All samples were prepared in a cold room at 4°C. Aβ concentration was obtained by tyrosine absorption at 276 nm using an extinction coefficient of 1390 cm^−1^ M^−1^.

MO extracts were added to Aβ solution to obtain the designed concentrations of both components. Aqueous extracts or water were added in a ratio 1/3, so the final composition of solutions in each sample was 33 mM sodium phosphate buffer with 13 mM of NaCl.

### 2.7 ThT Spectrofluorometric Measurements

ThT fluorescence emission assays were used to reveal the appearance of amyloid β‐sheet structure characterizing the conversion from disordered monomeric Aβ peptide to amyloid fibrils (LeVine 1999). ThT signal was monitored by using a 96‐well Thermo Scientific Fluoroskan Ascent F2 Microplate with thermostatic control at 37°C. ThT final concentration was 12 μM for all samples investigated. The excitation and emission wavelengths were 450 and 485 nm, respectively. All measurements were performed in duplicate.

### 2.8 Circular Dichroism Measurements (CD)

CD measurements were acquired by using a JASCO J‐815 CD Spectrometer. In particular, spectra for samples at different time were recorded at 20°C using a quartz cell with 0.2 mm path length. The appropriate blank contribution was subtracted.

### 2.9 Atomic Force Microscopy (AFM) Scans

AFM measurements were performed by using a Nanowizard III (JPK Instruments, Germany) mounted on an Eclipse Ti (Nikon, Japan) inverted optical microscope. Aliquots of samples were deposited onto freshly cleaved mica surfaces (Agar Scientific, Assing, Italy) and incubated for 20 min before rinsing with deionized water and drying under a low‐pressure nitrogen flow. Imaging of the samples was carried out in intermittent contact mode in air by using a NCHR silicon cantilever (Nanoworld, Switzerland) with a nominal spring constant ranging from 21 to 78 N/m, and typical resonance frequency ranging from 250 to 390 kHz.

### 2.10 Static and Dynamic Laser Light Scattering Measurements (SLS & DLS)

Light scattering experiments were carried on MO extracts, directly filtered in a quartz cylindrical cuvette (0.2 μm LG millex filter). Measurements were performed at 20°C on a Brookhaven Instrument BI‐9000 goniometer, equipped with a 50 mW He−Ne laser with wavelength *λ*_0_ = 632.8 nm. The intensity of the scattered light, I(t), and the intensity autocorrelation function, $g_{2}\left(t\right)$, were measured simultaneously by using a Brookhaven BI‐9000 correlator. Measurements were taken at the angle θ = 90°, corresponding to a scattering vector q=4πnλ0sinθ2=18.7μm−1, where n is the refractive index of the solution and λ_0_ is the in vacuo laser wavelength. The field autocorrelation function $g_{1}\left(t\right)=\sqrt{\left(g_{2}\left(t\right)-1\right)}$, was obtained from the measured $g_{2}\left(t\right),$ and analyzed by using a smoothing constrained regularization method (Stepanek 1993), in order to determine the distribution $A\left(\Gamma \right)$ according to:

(2) $$g_{1}\left(t\right)=\int A\left(\Gamma \right)e^{-\mathrm{\Gamma t}}d\Gamma$$

where $A\left(\Gamma \right)$ represents the contribution to the $g_{1}\left(t\right)$ amplitude, due to the species with diffusion coefficient D, considering that $\Gamma =Dq^{2}$. Then, by applying the Stokes−Einstein relation, $D_{h}=\frac{\mathrm{KT}}{3\mathrm{\pi \eta D}}$, from the diffusion coefficient distribution $A\left(D\right)$ the intensity weighted hydrodynamic diameter distribution $A\left(D_{h}\right)$ is obtained.

### 2.11 Cell Culture

The SH‐SY5Y (American Type Culture Collection, CRL‐2266) were cultured as monolayer and grown in 100 mm diameter polystyrene dishes using Dulbecco**'**s Modified Eagle's Medium (DMEM; Corning, New York, NY, USA) supplemented with 10% fetal bovine serum (FBS), 100 U/mL penicillin, and 100 μg/mL streptomycin (Corning). Cells were maintained in a humidified incubator at 37°C with a 5% CO_2_ atmosphere.

To differentiate the SH‐SY5Y cells to a neuron‐like state we used 10 μM all‐trans retinoic acid (RA). Before performing the experiments, cells were cultured for 7 days in the presence of RA, which was freshly added to the cell culture medium every 2 days (Piccirillo et al. 2018).

### 2.12 SH‐SY5Y Cell Treatments

RA‐differentiated SH‐SY5Y cells were treated for 24 h with GA at the concentration of 1 mM. GA is a glycolysis inhibitor that mediates the formation of neurotoxic agents known as advanced glycation end products (AGEs) (Ko et al. 2015; Ko, Lin, Lin, and Chang, 2010; Takeuchi et al. 2000). Our previous findings showed that GA can cause numerous AD‐like alterations that lead to cell death (Magi et al. 2020, 2021; Piccirillo et al. 2022; Preziuso et al. 2023). These changes include increased levels of AD markers (both Aβ_1–42_ and the hyperphosphorylated form of Tau protein) and the overproduction of mitochondrial ROS. Based on the above, GA was used to stimulate the accumulation of Aβ_1–42_ endogenously and then to assess the potential protective effect of RTE against its deleterious effects. Specifically, cells were pre‐exposed to RTE for 1 h at the final concentration of 4 μg/mL polyphenols and then treated with GA (1 mM) for 24 h (without withdrawing RTE). At the end of the experimental protocol, cells were collected for subsequent analysis.

### 2.13 Cell Viability Assays

To evaluate the viability of SH‐SY5Y cells, we used the 3‐(4,5‐dimethylthiazol‐2‐yl)‐2,5‐diphenyltetrazolium bromide (MTT) assay. This widely used colorimetric technique assesses cell metabolic activity by measuring the mitochondria's ability to metabolize MTT and produce insoluble purple formazan crystals (Piccirillo et al. 2020). Briefly, 60,000 cells/well were plated on a 12 well plate, cultured for 7 days in the presence of RA, and then subjected to the experimental protocol. At the end of the GA treatment, 500 μL of MTT solution (0.5 mg/mL in PBS) were added to each well. The multiwell plate was then incubated in a dark environment at 37°C and 5% CO_2_ for 1 h. Following the incubation, the formazan crystals were dissolved in dimethyl sulfoxide (DMSO). The amount of formazan produced is directly related to the cells' mitochondrial activity, and we measured the absorbance at a wavelength of 540 nm using a Victor Multilabel Counter plate reader from Perkin Elmer (Waltham, MA, USA). The results were expressed as a percentage relative to the control value.

### 2.14 Assessment of Mitochondrial ROS Production in SH‐SY5Y Cells

The quantitative analysis of ROS production in mitochondria of live cells was assessed by using the specific fluorescent probe MitoTracker CM‐H2XRos (Invitrogen Life Technologies, Carlsbad, CA, USA), following established procedures (Piccirillo et al. 2020). In brief, RA‐differentiated SH‐SY5Y cells were seeded on glass coverslip at the density of 100,000 cells/well and then were subjected to the specific treatments. At the end of the experimental procedure, cells were loaded with 300 nM of the dye for 30 min at 37°C, followed by three washes with PBS. Live cell imaging of mitochondrial ROS was performed by using an inverted Zeiss Axiovert 200 microscope (Carl Zeiss, Milan, Italy). CM‐H2XRos was excited at 560 ± 10 nm, and its emission was measured at 620 ± 20 nm. Images were captured at 5 s intervals, and the baseline ROS levels were observed for approximately 200 s. Subsequent analysis of fluorescence intensity was carried out offline post‐image acquisition, and the fluorescence values were expressed as percentages relative to the control value.

### 2.15 Immunocytochemistry

RA‐differentiated SH‐SY5Y cells were seeded on glass coverslip at the density of 100,000 cells/well and then treated with RTE and GA as previously described. At the end of the experimental protocol, cells were loaded with 300 nM MitoTracker (MitoTracker Red CMXRos M7512, Invitrogen) for 30 min at 37°C. This treatment aimed to stain the mitochondria within live cells to track them. After that, the cells were fixed with 3.7% formaldehyde for 30 min at room temperature (RT). Subsequently, cells were permeabilized with Triton X‐100 for 5 min at RT and then incubated for 1.5 h with Aβ_1–42_ primary antibodies (mouse monoclonal IgG1antibody clone 12F4, Cat. 805,501, Biolegend, San Diego, CA, USA, dilution 1:100 in PBS with 1% BSA). To detect the immunoreaction, a conjugated secondary antibody, specifically Alexa Anti‐Mouse 488 (Catalog A11059, Thermo Scientific) diluted at 1:200, was employed (Piccirillo et al. 2022). Analysis of fluorescence intensity was performed offline after image acquisition. Fluorescence values were reported as percentages of the control value.

### 2.16 Statistical Analysis

SH‐SY5Y data errors are expressed as the standard error of the mean. Values less than 0.05 were considered statistically significant. Differences among means were assessed by one‐way ANOVA followed by Dunnett's post hoc test. Statistical comparisons were performed using GraphPad Prism 5 software (GraphPad Software Inc., San Diego, CA, USA).

## 3 Results

### 3.1 Characterization of MO Extracts

#### 3.1.1 Anti‐Oxidative Capacity and TPC of the Extracts

Water at room or boiling temperature was utilized as a green solvent to extract bioactive compounds from MO dry leaves. The room‐ and high‐temperature water extracts, RTE and HTE respectively, were analyzed for their antioxidant capacity, TPC and chemical composition. To appreciate the validity of these green extractions, the same analysis was performed as a reference for a sample obtained by using for the extraction a 50% ethanol‐water mixture (ME). The antioxidant capacity was evaluated by using the ORAC assay. The obtained values are reported in Table 1. The TPC values were obtained by using the Folin–Ciocâlteu assay. The TPC value for 1 g of MO dry leaves is expressed as mg of GAE. Extraction procedures affect the indexes evaluated, as reported in Table 1. High temperature extraction indexes get very close to the ones obtained in case of ME, differently from the ones obtained for room temperature procedure.

**TABLE 1:** TPC and ORAC values of MO extracts.

	TPC mg GAE/g dry leaves	ORAC value μmol TE/g dry leaves
HTE	23.7 ± 0.1	49 ± 2
RTE	16.4 ± 0.2	33 ± 1
ME	28.3 ± 0.1	55 ± 2

Abbreviations: GAE, gallic acid equivalent; MO, *Moringa oleifera* ; ORAC, oxygen radical absorbance capacity; TPC, total phenol content.

#### 3.1.2 UHPLC‐HESI‐MS Analysis of MO Extracts

**FIGURE 1:**
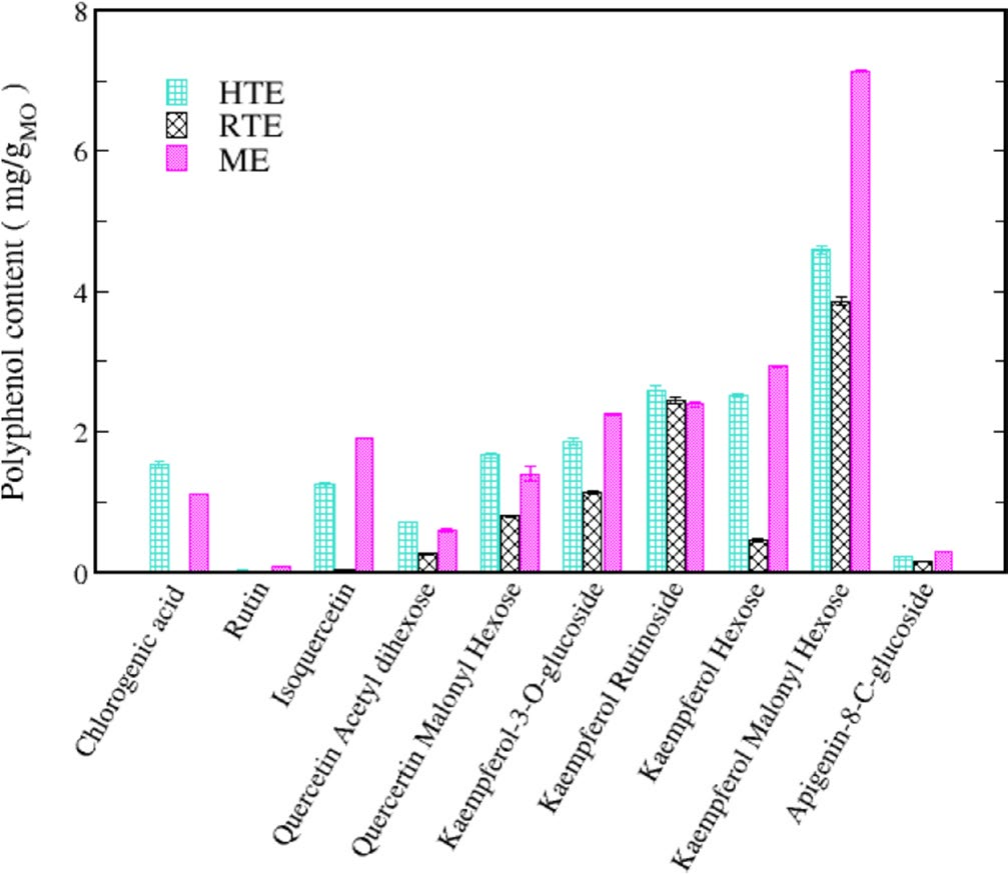
Comparison between polyphenol content in HTE (cyan) RTE (black) and ME (pink) as determined by UHPLC‐HESI‐MS analysis. Data are expressed as mg of polyphenols *per* g of *Moringa oleifera* (MO) dry leaves.

The polyphenols composition of the MO extracts was detected by UHPLC‐HESI‐MS analysis. The extraction procedures influence composition and relative amount of the detected compounds in the three MO extracts as evidenced in Figure 1. In particular, the quantifiable polyphenols accounts for 16.98 ± 0.12, 9.15 ± 0.09 and 20.175 ± 0.11 mg per MO dry leaves respectively in HTE, RTE, and ME (see Table S1). The composition of HTE closely resembles that of ME, although the total amount of measurable polyphenols is 15% less than ME. However, the results highlighted that some components, such as chlorogenic acid (1.530 mg/g) and kaempferol rutinoside (2.590 mg/g), were more concentrated in the HTE compared to the ME. Distinctively, the RTE is primarily free of chlorogenic acid and overall is less concentrated, except for kaempferol rutinoside (2.44 mg/g).

### 3.2 MO Water Extracts Effect on Aβ_1‐42_ Fibrillation

**FIGURE 2:**
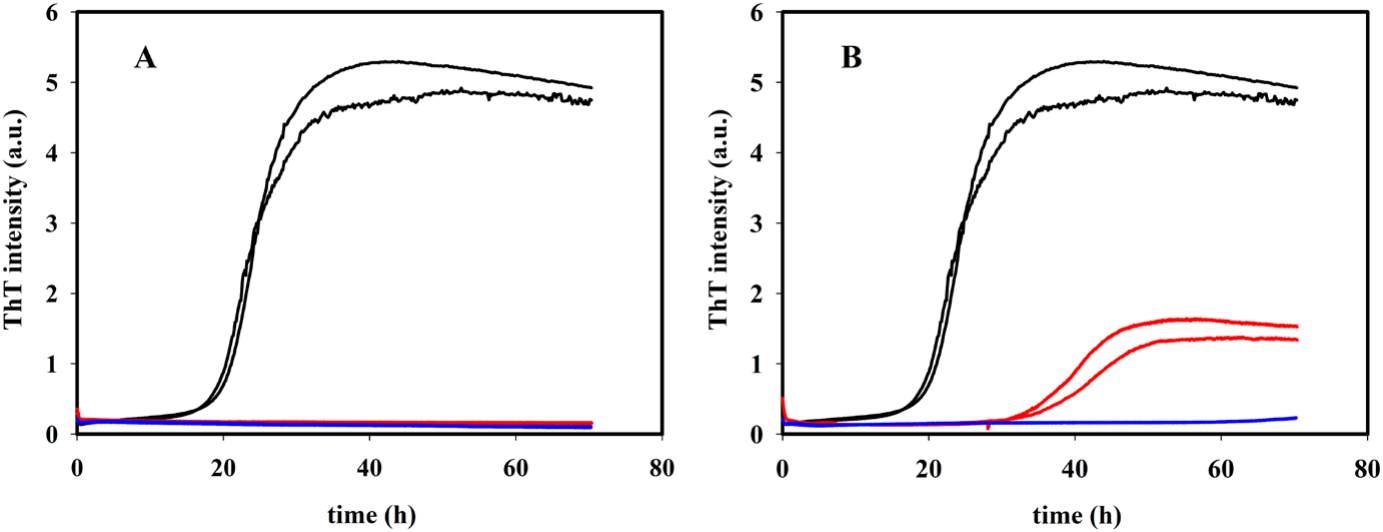
Inhibitory activity of *Moringa oleifera* (MO) water extracts on Aβ_1–42_ aggregation. (A) Comparison between fibrillation kinetics of 15 μM Aβ_1–42_ alone (black line) and 15 μM Aβ_1–42_ in the presence of RTE at different TPC value (82 μg/mL gallic acid equivalent (GAE) red line and 24 μg/mL GAE blue line); (B) Comparison between fibrillation kinetics of 15 μM Aβ_1–42_ alone (black line) and in presence of HTE at different TPC value (118 μg/mL GAE red line and 7.1 μg/mL GAE blue line).

The anti‐amyloidogenic effect of both aqueous HTE and RTE on Aβ_1–42_ fibrillation kinetics was studied by ThT fluorescence assay. ThT fluoresces when interacts with β‐sheet structure regions, so allowing to appreciate Aβ_1–42_ conversion from random coil to β‐sheet structure that precedes the process of fibrils growth. As shown in Figure 2, when Aβ_1–42_ is incubated at 37°C, ThT fluorescence signal follows a characteristic sigmoidal curve, with a lag‐phase of about 18 h, reaching a plateau after about 40 h. To test the efficiency of MO extracts, the less rich on polyphenol RTE was diluted 3 and 10×, so obtaining a concentration of 82 and 24.6 μg equivalent GA per ml respectively. HTE, the richer sample, was diluted 3 and 50×, so reaching a polyphenol concentration of 118 and 7.1 μg equivalent GA per ml respectively. When Aβ_1–42_ is incubated with RTE (Figure 2A) no increase in the ThT fluorescence was observed for both dilutions in the experiment time window observed. When incubated with HTE, surprisingly, only the sample with the highest polyphenols concentration showed a ThT signal increase, though a lag‐phase about twice longer was observed with respect to the Aβ_1–42_ alone and a lower ThT emission plateau value was reached.

**FIGURE 3:**
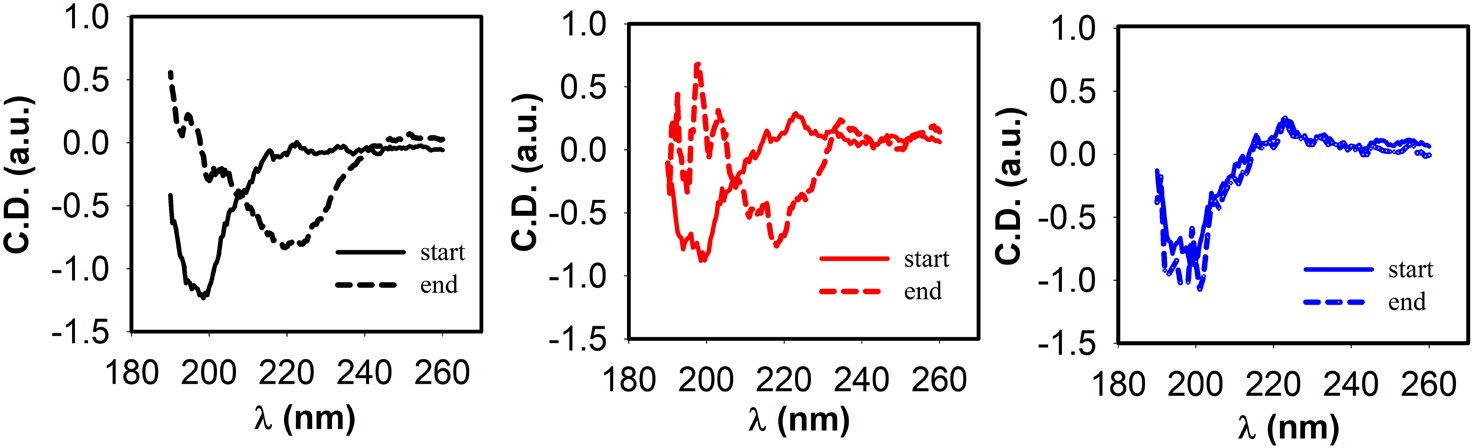
Effect of *Moringa oleifera* (MO) water extracts on Aβ_1–42_ secondary structure variation during fibrillogenesis. Far‐UV CD spectra at the start (continuous lines) and at the end (72 h, dashed lines) of the aggregation kinetics for Aβ_1–42_ alone (black lines), and in the presence of HTE at 118 μg/mL gallic acid equivalent (GAE) concentration (red lines) or RTE at 82 μg/mL GAE concentration (blue lines).

To exclude any interference by ThT and polyphenols and confirm the absence of β‐sheet structure for sample with flat ThT, the secondary structure of peptides was investigated by far‐UV CD, too. Spectra of samples at the beginning and at the end of the kinetics (72 h) were recorded (Figure 3). Changes from random coil to β‐sheet were observed for the Aβ_1–42_ and Aβ_1–42_/HTE samples at highest concentration, whereas the sample Aβ_1–42_/RTE at highest concentration did not show any structural change after 72 h of incubation. Lastly, AFM images were acquired to identify the objects on the nano−/micro‐meter scale present in the same samples at the final stage of reaction.

**FIGURE 4:**
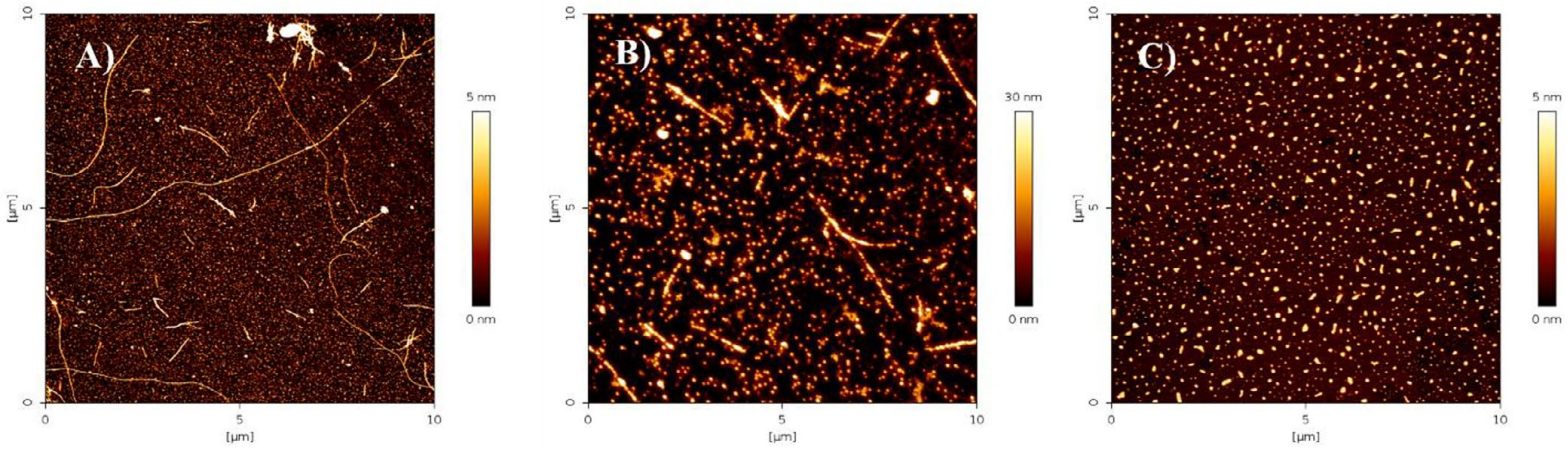
Representative atomic force microscopy (AFM) scans of samples at the end of the kinetics of aggregation for Aβ_1‐42_ alone (A), and in the presence of HTE at 118 μg/mL gallic acid equivalent (GAE) concentration (B) or at 82 μg/mL GAE concentration (C).

In Figure 4, representative AFM scans for Aβ with and without RTE and HTE at higher concentration are reported. Fibers are present in the solution of Aβ_1–42_, small elongated aggregates are also present in the Aβ_1–42_/HTE sample, while no fibers are detected in the Aβ_1–42_/RTE sample. Interestingly, more spherical objects appeared in both samples with MO extracts (with dimensions on the order of tens of nanometers).

### 3.3 Structural Characterization of MO Extracts

To unravel if the spherical objects revealed by AFM measurements are present in the MO extracts alone or arise from the interaction with the Aβ peptide, and to investigate their potential involvement in the inhibitory effect on Aβ_1–42_ fibrillation, a structural characterization of the MO extracts alone was performed.

**FIGURE 5:**
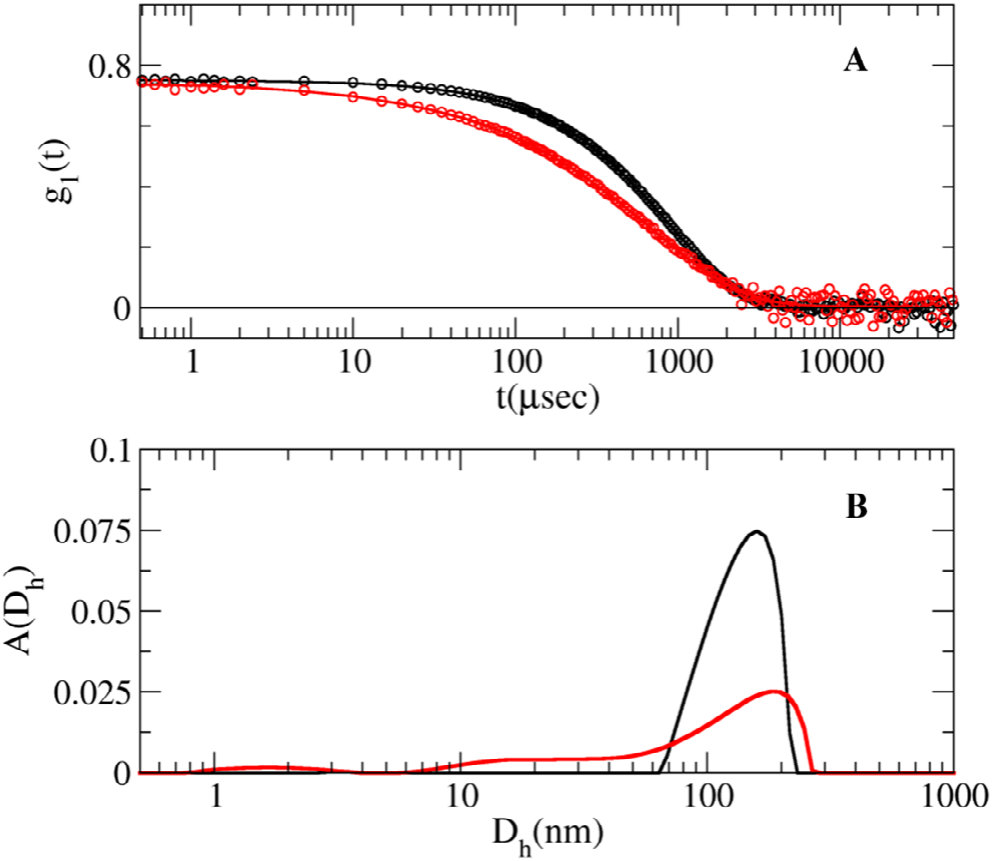
(A) Field autocorrelation function for HTE (black circles) and RTE (red circles) samples. Solid lines represent the data fits. (B) Intensity weighted size distribution obtained by the analysis of the field autocorrelation function for HTE (black) and the RTE (red) sample, according to the expression reported in section 2.

Comparative DLS measurements performed on HTE and RTE are showed in Figure 5. From the analysis of the field autocorrelation function the presence of ⁓100 nm wide aggregates for both samples can be inferred. In particular, for the HTE sample only these particles (D⁓100 nm) can be detected, while for the RTE sample the size distribution strongly suggests also the presence of two different populations of smaller aggregates. In addition, the abundance of smaller aggregates in RTE is also supported by the static light scattered intensity measured for the two samples, being one order of magnitude less for RTE with respect to HTE. The large presence of 100 nm species in the HTE sample does not exclude the presence of smaller aggregates, whose signal is simply covered by the scattering from the larger species. In fact, in the RTE sample this detection is possible, due to the abundancy of the small aggregates with respect to the larger ones. The distribution of species according to number concentration would indeed revert the areas relative to the species.

**FIGURE 6:**
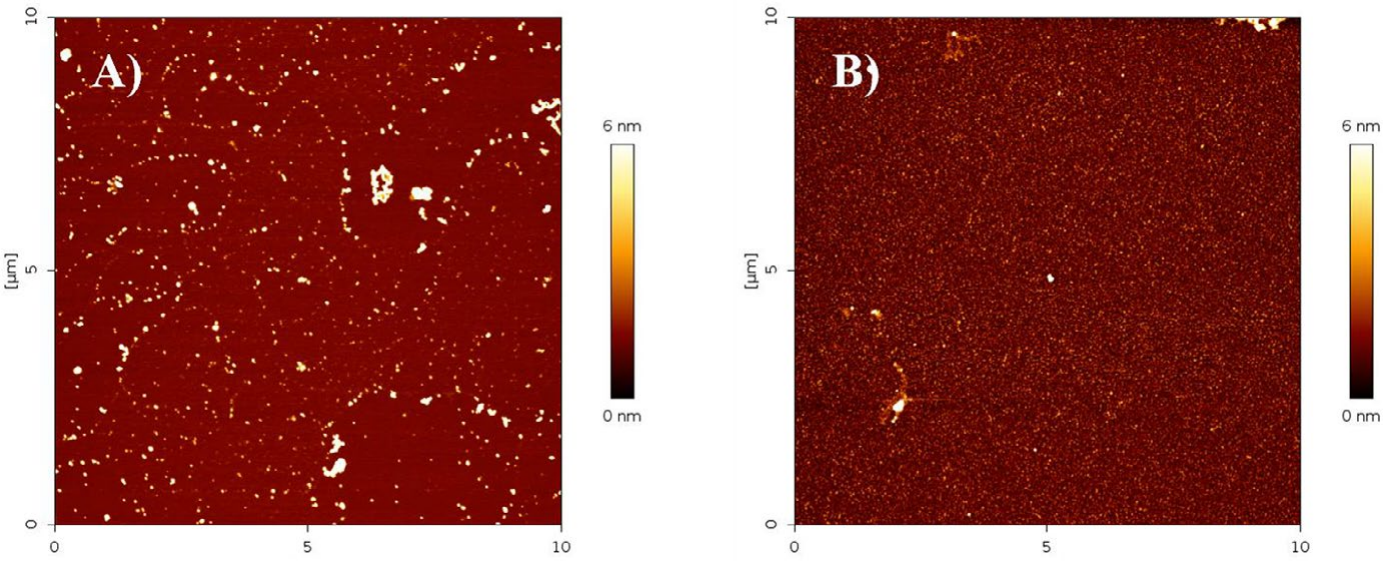
Representative atomic force microscopy (AFM) scans of HTE (A) and RTE (B) extracts, deposited on mica and dried.

In Figure 6 representative AFM scans, acquired for the samples of MO extracts characterized by DLS, are reported. AFM characterization highlights the presence in the HTE sample of aggregates with tendency to interact each other, organized as linear chains, although not connected. In the RTE sample a carpet of aggregates of smaller dimensions appears. These results are consistent with DLS results, highlighting that polyphenol aggregates are intrinsic in MO water extracts.

**FIGURE 7:**
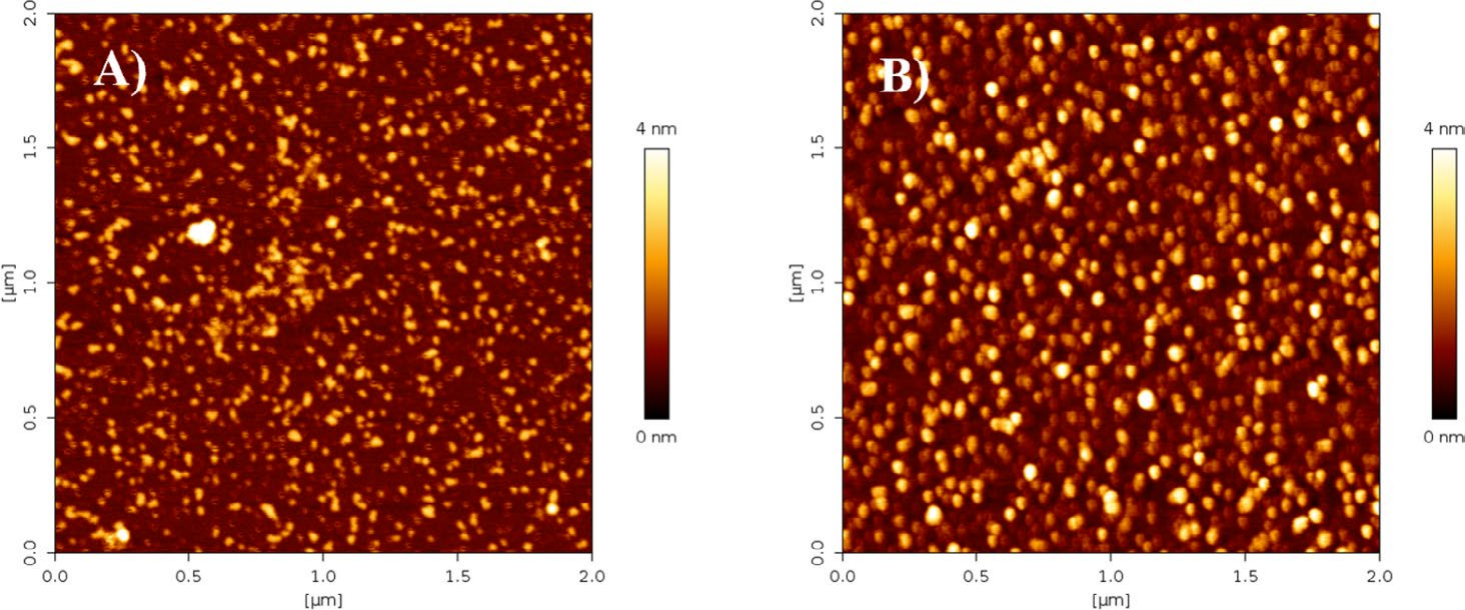
Representative atomic force microscopy (AFM) scans of HTE (A) and RTE (B) extracts diluted 60 times, deposited on mica and dried.

Interestingly, AFM measurements of both RTE and HTE samples, obtained by diluting 60 times the extracts before deposition on mica highlight the presence in both the samples of a regular background made of aggregates apparently tens of nanometers wide (Figure 7). Consistently, DLS analysis for the RTE sample allowed to detect these small aggregates, while was blind to them for the HTE sample.

### 3.4 Protective Effect of MO Water Extracts on LAN5 Cells Against Oxidative Stress Induced

We first determined the effect of 24 h of exposure to RTE or HTE polyphenol concentrations ranging from 0.125 to 16 μg/mL on LAN5 neuroblastoma cells. As shown in Figure S1, no toxicity was detected at all concentration tested. Moreover, a significant gradual increase in cell viability was observed at higher concentration of RTE.

We chose to use the 2 μg/mL concentration to investigate the ability of RTE and HTE to counteract the oxidative stress on LAN5 cells by using the tert‐Butyl hydroperoxide (TBH), a direct‐acting oxidative stress‐inducing agent. Cell viability considerably decreased after treatment with 0.125 mM of TBH for 1 h compared to the control (Ctl). Conversely, when cells were pretreated with RTE or HTE a cytoprotection was detected (Figure S2A) and a decreasing of ROS induced by TBH was detected (Figure S2B). Overall, the results suggest that water extracts from MO, not only enhance cell viability but also possess antioxidant properties, offering protection against oxidative stress‐induced damage in LAN5 neuroblastoma cells.

### 3.5 Protective Effect of RTE in an AD‐Like Cell Model of SH‐SY5Y Against Mitochondrial Oxidative Stress

**FIGURE 8:**
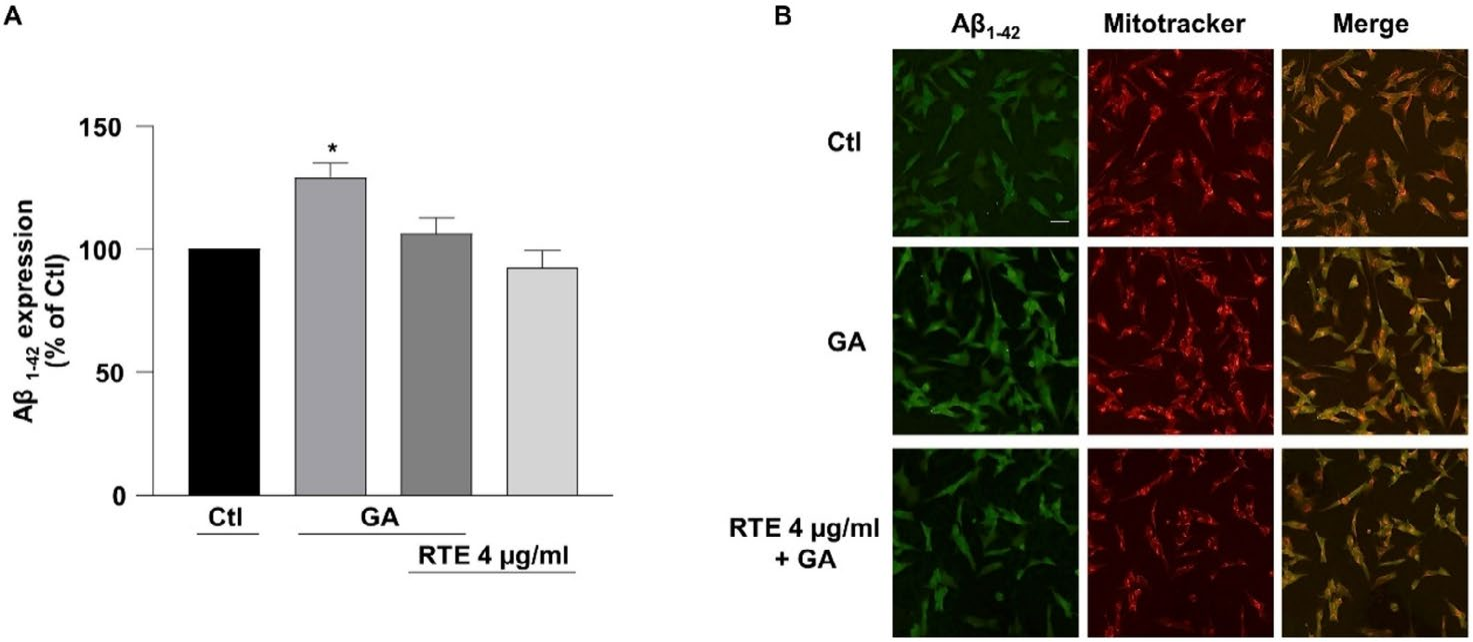
Effect of RTE on Aβ_1–42_ expression in retinoic acid‐differentiated (RA) SH‐SY5Y cells challenged with glyceraldehyde (GA). (A) Quantitative analysis of Aβ_1–42_ levels, and (B) representative images of Aβ‐amyloid expression in cells exposed to RTE (4 μg/mL GAE) for 1 h and then treated with GA (1 mM) for 24 h (without withdrawing RTE). Aβ_1–42_ expression was evaluated by immunofluorescence staining. Differences among means were evaluated by one‐way ANOVA followed by Dunnett's post hoc test. In each experiments the intensity of fluorescence was expressed as a percentage of the control value. Scale bar 50 μm. *Significant versus all groups (*p* < 0.01 vs. control groups, *p* < 0.05 vs. RTE + GA); *n* = 4. GAE, gallic acid equivalent.

**FIGURE 9:**
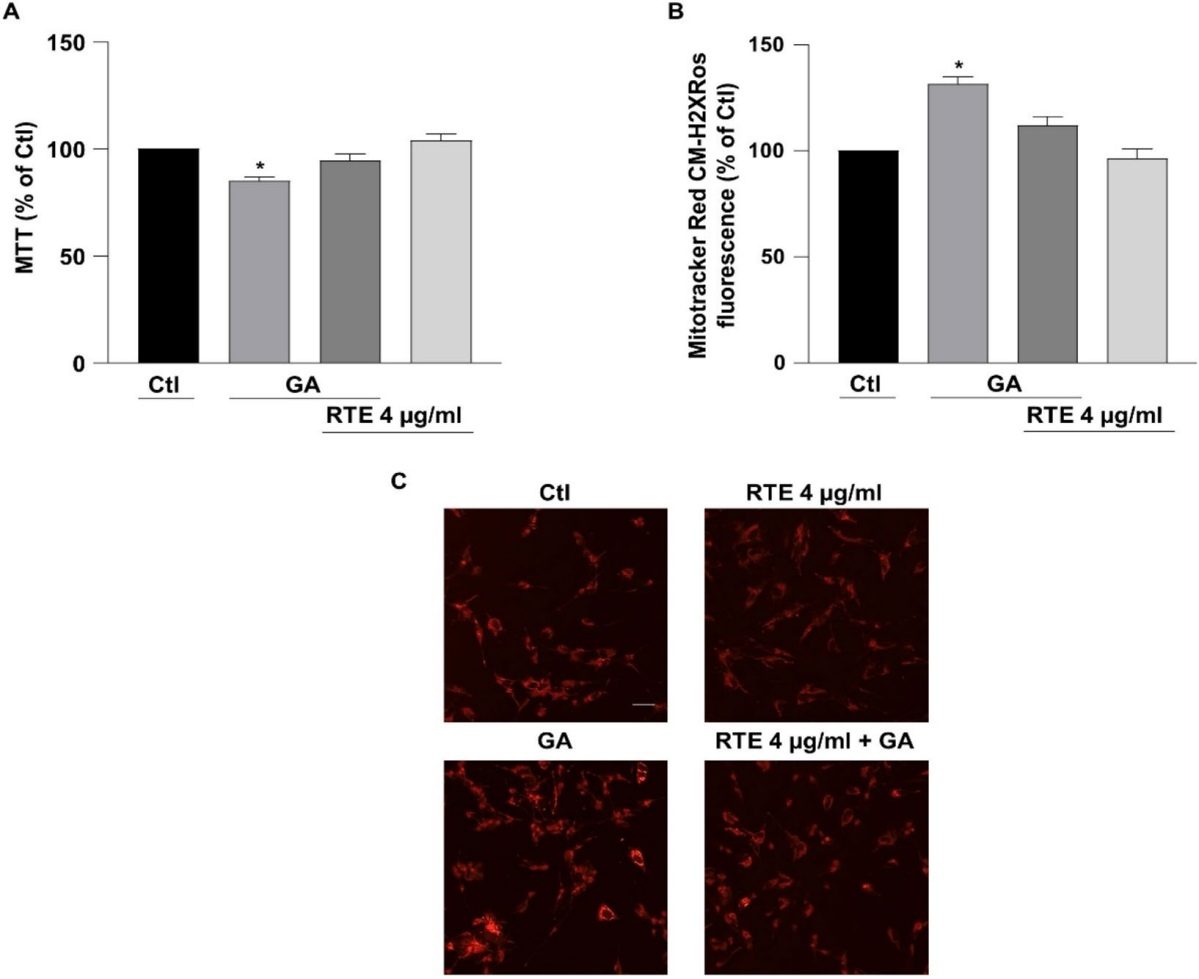
Effect RTE on cell viability and mitochondrial ROS production in retinoic acid‐differentiated (RA) SH‐SY5Y cells challenged with glyceraldehyde (GA). Cells were exposed to RTE 4 μg/mL for 1 h and then treated with GA (1 mM) for 24 h (without withdrawing RTE). (A) Cells viability was evaluated by MTT assay. (B) Mitochondrial ROS generation assessed by measuring MitoTracker Red CM‐H2XRos fluorescence intensity. (C) Representative images of mitochondrial ROS productions. Differences among means were evaluated by one‐way ANOVA followed by Dunnett's post hoc test. In each experiments the reduction of MTT and the intensity of MitoTracker Red CM‐H2XRos fluorescence were expressed as percentage of the control values. Scale bar 50 μm. (A) *Significant versus all groups (*p* < 0.0001 vs. control groups, *p* < 0.05 vs. RTE + GA); *n* = 7. (B) *Significant versus all groups (*p* < 0.001 vs. Ctl, *p* < 0.0001 vs. RTE, *p* < 0.01 vs. RTE + GA); *n* = 4.

The protective effects of MO extract obtained at room temperature versus the typical cell alterations observed in AD were evaluated on retinoic acid‐differentiated SH‐SY5Y cells challenged with GA. Consistent with previous studies, the glycolysis inhibitor GA is able to reproduce hypometabolism condition along with mitochondrial alterations and oxidative stress, events that are frequently observed at the early stage of AD, also including the deposition of Aβ_1‐42_ and the overproduction of ROS (Magi et al. 2020, 2021; Piccirillo et al. 2022; Preziuso et al. 2023). To explore the protective effect of RTE in this experimental setting, RA‐differentiated SH‐SY5Y cells were subjected to a pre‐treatment of 1 h with RTE at the concentration of 4 μg/mL followed by 24 h exposure to GA. Given that GA induced the accumulation of Aβ_1–42_ within mitochondria (Piccirillo et al. 2022), firstly we investigated the effect of RTE on the increase in Aβ_1–42_ deposition induced by GA exposure. Results indicate that MO extract significantly reduced Aβ_1–42_ accumulation compared to cells exposed to GA in the absence of it (Figure 8). Moreover, as shown in Figure 9A, pre‐exposure to RTE significantly ameliorated cell viability after 24 h of GA treatment.

Finally, to analyze whether the reduction of Aβ_1–42_ and the enhancement of cell survival induced by MO (Figure 9A) could also be related to its potential antioxidant effect, the production of mitochondrial ROS was measured: RTE mitigated the increase in mitochondrial ROS triggered by GA (Figure 9B), suggesting that its potential antioxidative mechanism could contribute to limit the cell injury induced by GA.

## 4 Discussion and Conclusions

In this work water extracts of MO leaves at room temperature (RTE) and at boiling temperature (HTE) are compared by chemical composition, antioxidative/antiradical properties and antifibrillogenesis action on Aβ_1–42_ peptide. Due to the clear results obtained for both the extracts, but especially for RTE, as inhibitors of Aβ_1–42_ amyloid aggregation, the effect of a pre‐treatment with RTE is evaluated on retinoic acid‐differentiated SH‐SY5Y cells, a cellular model of AD, challenged with GA.

In the last decades, plants and their extracts have received great attention due to their beneficial effect on health. To improve the efficiency in the recovery phytochemicals extractions are often performed with organic solvents, noxious for health and environment, and with the use of microwave or high pressure (Daghaghele et al. 2021; Gunalan et al. 2022, 2023). Interestingly, less eco‐impactful and cheaper strategies have shown interesting results related to the yield and following use of extracts (García‐Beltrán et al. 2020; Hamany Djande et al. 2018; Sandanuwan Kirindage et al. 2022; Silva et al. 2022). In this context we have chosen water at room and boiling temperature to perform the extraction from MO leaves. To validate the extraction procedure, we have performed a comparative extraction by an organic solvent (ethanol 50%) (Berhanu and Masunov 2010; Ono et al. 2003; Rana et al. 2022; Rudrapal et al. 2022). Interestingly, as reported in Table 1 and Figure 1, it is evident that a fast and inexpensive extraction with water at 100°C leads to a good compromise between yield and cost. In fact, ORAC and TPC values obtained for the HTE are 12% and 16% lower than the respective values obtained for ME. Furthermore, UHPLC‐HESI‐MS analysis shows that phenolic compounds in HTE have a 15% lower concentration than in ME. Some components, however namely chlorogenic acid and kaempferol rutinoside, are more abundant in HTE than in ME and RTE.

Clearly, water high temperature improves the yield: ORAC and TPC values for RTE are lower than those for HTE by 33% and 31%, respectively. Regarding UHPLC‐HESI‐MS analysis, all components present in the RTE are less concentrated than in the other extracts except for Kaempferol Rutinoside, which is slightly more abundant in RTE with respect to ME. Although the RTE contains lower amounts of polyphenols with respect to the other extracts, the presence of Kaempferol and Quercetin derivatives, and in general of all the polyphenols here detected, suggests the healthy potential of the extracts on neurodegenerative diseases, in general, and specifically on AD (Berhanu and Masunov 2010; Hanaki et al. 2016; Ono et al. 2003; Stefanescu et al. 2020). We focused our characterization on polyphenolic compounds, aware of the complexity of the vegetal extracts which may contain traces of various molecules, such as ions or vitamins or other co‐solvents. While the potential synergistic effects of other substances within the extracts cannot be ruled out, the beneficial effects of our extracts on fibrillation inhibition and protection against ROS‐induced damage can be attributed primarily to the polyphenolic compounds, as confirmed by numerous studies demonstrating the protective effect of individual polyphenols or their mixtures (Berhanu and Masunov 2010; Ono et al. 2003; Rana et al. 2022; Rudrapal et al. 2022). Different and complex mechanisms control the reduction of toxic aggregates and the ROS protective action (Bhat et al. 2022; Borana et al. 2014; Obulesu and Rao 2011; Tinku and Choudhary 2023). AD is a neurodegenerative disease characterized by a complex pathological pathway where the oligomers, which precede the formation of well‐ordered β‐sheets, are believed to be the most toxic species, responsible for the cell membranes damage and for mitochondrial ROS generation (Canale et al. 2013; Haass and Selkoe 1993, 2007; Kayed and Lasagna‐Reeves 2013; Mangione et al. 2015; Matsuzaki 2014; Pike et al. 1993; Ricci et al. 2019). Thus, polyphenols, capable of acting as inhibitors of oligomeric aggregation and as protective agents, can attend with a double protective action. Polyphenols can limit the aggregation by interacting with peptide regions in the sequence that are crucial in the aggregation pathway. Boubakri et al. (2020) showed that Kaempferol, by hindering amyloid fibrils formation, reduced Aβ_1–42_ cytotoxicity in human neuroblastoma cells, SH‐SY5Y. The neuroprotective action was correlated with inhibition of peptide aggregation and in particular with a reduction of its binding to the cell membrane. Moreover, Kaempferol counteracted the oxidative stress on cell thus enhancing neuroprotective effect. Similarly, Sharoar et al. (2012) showed beneficial anti‐amyloidogenic properties of Kaemperol 3‐O‐glucoside by the inhibition of the fibrillation propensity and by originating off‐pathway non‐amyloidogenic not cytotoxic conformational structures of amyloid‐beta peptide. Beg et al. (2018) evidenced the protective effect of Kaempferol on the transgenic drosophila model of Alzheimer's disease, by revealing that the exposure of AD flies to kaempferol delayed the loss of climbing ability, memory, reduced the oxidative stress and acetylcholinesterase activity. Some reviews are dedicated to the role of quercetin on attenuating Alzheimer's disease by limiting the Aβ toxic fibrillation pathway or by its antioxidative and anti‐inflammatory properties (Khan et al. 2020; Zaplatic et al. 2019). Noteworthy, polyphenolic compounds present in aqueous MO extracts have been considered neuroprotective against Alzheimer's disease by exerting modulation of the activity of cholinergic and purinergic enzymes related to neuroinflammatory processes in microglial cell (Adefegha et al. 2021). In our study, the protective effect of MO water extracts against oxidative stress induced in LAN5 neuroblastoma human cell line and in particular the beneficial health properties of RTE on an AD cell model has been demonstrated. In fact, experiments on RA‐differentiated SH‐SY5Y cells exposed to GA show a significant reduction in intracellular Aβ_1–42_ deposition and mitochondrial ROS formation triggered by GA, when pretreated with the RTE extract. Moreover, both water extracts show a very strong inhibiting effect on in vitro Aβ fibrillogenesis. Very interestingly, a powerful inhibitory effect is observed at very low concentration of RTE (Figures 2, 3, 4) and no dose dependence is observed, being the extract fully active at all the studied concentrations. Undoubtedly, also HTE has a strong inhibitory effect on Aβ in vitro aggregation. Characterization of MO extracts alone made it possible to understand that in HTE sample a higher number of large aggregates is present. This result allows us to interpret why the higher is the concentration of HTE the less efficient is its inhibition effect (Figure 2B). In this case in fact, though at high concentration the inhibition is anyway strongly present, with a considerable delay on the lag phase together with a reduction in the final number of fibrils compared to the Aβ sample alone, probably the presence of such large aggregates favors stochastic local nucleation and consequent fibrils growth, as evidenced from ThT experiments. For this sample, CD spectra and AFM scans confirm the final β‐sheet structure conversion and fibers formation, respectively (Figures 3B and 4B). Interestingly, AFM scans, characterizing the final aggregation stage, evidence the presence of different kind of aggregates in both samples treated with MO water extracts (Figure 4B,C). Taken into consideration all the collected results, we consider that polyphenols can form supramolecular aggregates of various size by simply self‐assembling, process probably facilitated during the extraction by the high concentration of bioactive compounds, as well as by the presence of ions and/or proteins, though HPLC characterization (data not shown) could not detect any typical protein signal. In the last years a plethora of scientific articles highlighted the extraordinary capacity of polyphenols to self‐assemble realizing nanoparticle materials with different size, shape and chemical properties. Xiang et al. (2017) used a green synthetic strategy to prepare polyphenol nanoparticles by using theophylline as cross‐linker to drive the polymerization of tea polyphenols by a simple preparation protocol in water at 80°C. Ejima et al., produced films and microcapsules different in size and shape by the coordination of Fe^3+^ with natural polyphenols (Ejima et al. 2013). Debnath et al. prepared nanoparticles of epigallocatechin‐3‐gallate by adding polysuccinimide derivates (EGCG) (Debnath et al. 2016). All these approaches are based on the extraordinary chemical characteristics of polyphenols that thanks to their functional groups, the phenolic hydroxyl moieties, can interact through covalent or non‐covalent bonds (ionic, π‐π interactions, hydrogen bonding, hydrophobic interactions) to create supramolecular self‐assembly with different shape and dimension (Yin et al. 2024). Considering the tendency of polyphenols to interact with each other's, the results here presented coherently sustain that a self‐assembling process undergoes, due probably to the high molecular richness on the extracts. Moreover, static and dynamic light scattering experiments coupled with AFM characterization on HTE and RTE sample alone, confirm the presence of a large number of aggregates (about 100 nm) together with smaller ones (10 nm) in the HTE sample and of a prevalence of similar smaller aggregates in the RTE sample. The role of the polyphenol association grade could be complex in the inhibitory effect of Aβ peptide fibrillization: large aggregates (in HTE) could act as seeds and onset Aβ peptide fibrillization; the dilution of HTE leads to the reduction of such aggregates thus causing a more efficient inhibition process. Moreover, the presence of stable polyphenol assemblies, present in both extracts even at very strong dilutions, suggests that both free and aggregated polyphenols elicit an efficient inhibition mechanism, due to phenolic group ability to interact with aggregation prone, metastable species. In fact, the recruitment of so called protofibrillar species, highly toxic, strongly hinders the autocatalytic amyloid growth, as seen in other inhibition cases (Librizzi et al. 2014; Vilasi et al. 2019). A deeper study of these regular structures is beyond the scope of this paper and is the focus of another project aimed to explore biotechnological potentiality of water extracted MO polyphenols aggregates, by considering their high yield and their green extraction from a natural source.

For now, the present study points out that a green extraction with water both at boiling and room temperature is useful to obtain polyphenols‐rich extracts with a potent protective role against Aβ fibrillation in vitro. Additionally, this protective effect extends to an in vitro Alzheimer's disease‐like cell model treated with room temperature extracts. This reveals further aspects in the beneficial properties of *Moringa oleifera* , once again affirming the importance of polyphenols in contrasting the processes at the basis of neurodegeneration.

## Author Contributions

**Rita Carrotta:** conceptualization (equal), data curation (equal), investigation (equal), visualization (equal), writing – original draft (equal), writing – review and editing (equal). **Silvia Vilasi:** conceptualization (equal), data curation (equal), investigation (equal), visualization (equal), writing – original draft (equal), writing – review and editing (equal). **Maria Assunta Costa:** data curation (equal), investigation (equal), visualization (equal), writing – review and editing (equal). **Fabio Librizzi:** conceptualization (equal), data curation (equal), investigation (equal), visualization (equal), writing – original draft (equal), writing – review and editing (equal). **Vincenzo Martorana:** data curation (equal), investigation (equal), visualization (equal), writing – review and editing (equal). **Rosa Passantino:** data curation (equal), investigation (equal), visualization (equal), writing – review and editing (equal). **Carla Buzzanca:** investigation (equal), visualization (equal), writing – review and editing (equal). **Vita di Stefano:** data curation (equal), investigation (equal), visualization (equal), writing – review and editing (equal). **Maria Grazia Ortore:** investigation (equal), visualization (equal), writing – review and editing (equal). **Silvia Piccirillo:** investigation (equal), visualization (equal), writing – review and editing (equal). **Alessandra Preziuso:** investigation (equal), visualization (equal), writing – review and editing (equal). **Simona Magi:** data curation (equal), investigation (equal), visualization (equal), writing – review and editing (equal). **Maria Rosalia Mangione:** conceptualization (equal), data curation (equal), investigation (equal), supervision (lead), visualization (equal), writing – original draft (equal), writing – review and editing (equal).

## Ethics Statement

The authors have nothing to report.

## Conflicts of Interest

The authors declare no conflicts of interest.

## Supporting information


Data S1.

## Data Availability

The Data that support the findings of this study are available on request from the corresponding authors.
