# Supplementary material for: Moringa oleifera Extracts as Strategic Phyto‐Therapy for Alzheimer's Disease
Source: Food Sci Nutr. 2025 Mar 26;13(4):e70007. doi: 10.1002/fsn3.70007 (PMC11938381; doi:10.1002/fsn3.70007)
Supplement: Supplementary file 1 — Data S1. [file FSN3-13-e70007-s001.docx]

**SUPPORTING INFORMATION**

**Moringa Oleifera extracts as strategic phyto-therapy for Alzheimer’s disease**

**Rita Carrottaa* Silvia Vilasi,a* Maria Assunta Costaa, Fabio Librizzia**§**, Vincenzo Martoranaa, Rosa Passantinoa, Carla Buzzancab, Vita di Stefanob, Maria Grazia Ortorec, Silvia Piccirillod, Alessandra Preziusod, Simona Magid, and Maria Rosalia Mangionea**§

*aInstitute of Biophysics, National Research Council, Palermo, Italy;*

*bDepartment of Science and Biological, Chemical and Pharmaceutical Technologies STEBICEF, University of Palermo, Italy;*

*cDepartment of Life and Environmental Sciences, Marche Polytechnic University, Ancona, Italy;*

*dD Department of Biomedical Sciences and Public Health, School of Medicine, Marche Polytechnic University, Ancona, Italy;*

* These authors contributed equally to this work

§Corresponding author ([mariarosalia.mangione@ibf.cnr.it](mailto:mariarosalia.mangione@ibf.cnr.it), fabio.librizzi@ibf.cnr.it)

**Table S1**

Table 1 – Polyphenol quantitative determination, expressed as mg/g of MO dry leaves, of extracts obtained with three different solvents

|  | | | |
| --- | --- | --- | --- |
| *UHPLC-ESI-MS Quantitative determination* | | | |
|  | *HTE*  *mg/g MO dry leaves* | *RTE*  *mg/g MO dry leaves* | *ME*  *mg/g MO dry leaves* |
| *Chlorogenic acid* | *1.53 ± 0.04* | *--* | *1.109 ± 0.003* |
| *Rutin* | *0.024 ± 0.002* | *--* | *0.081 ± 0.001* |
| *Isoquercetin* | *1.244 ± 0.009* | *0.029 ± 0.001* | *1.919 ± 0.002* |
| *Quercetin Acetyl dihexose* | *0.715 ± 0.007* | *0.26 ± 0.01* | *0.60 ± 0.02* |
| *Quercetin Malonyl hexose* | *1.67 ± 0.04* | *0.80 ± 0.01* | *1.40 ± 0.10* |
| *Kaempferol-3-O-glucoside* | *1.86 ± 0.05* | *1.14 ± 0.02* | *2.253 ± 0.009* |
| *Kaempferol Rutinoside* | *2.59 ± 0.07* | *2.44 ± 0.05* | *2.39 ± 0.04* |
| *Kaempferol Hexose* | *2.52 ± 0.02* | *0.46 ± 0.03* | *2.93 ± 0.02* |
| *Kaempferol Malonyl Hexose* | *4.60 ± 0.06* | *3.86 ± 0.06* | *7.10 ± 0.02* |
| *Apigenin-8-C-glucoside* | *0.227 ± 0.003* | *0.161 ± 0.002* | *0.291 ± 0.001* |
| ***Sum*** | ***16.9 ± 0.1*** | ***9.1± 0.1*** | ***20.1 ±0.1*** |
|  | | | |

**Figure S1**

Fig. S1 - Effects of RTE and HTE on LAN5 cell viability. LAN5 cells were treated with different concentrations of RTE or HTE for 24 h. Cell viability was evaluated by MTS assay and expressed as percentage relative to the control (Ctl). The data are shown as means ± SD of three separate experiments at least

**
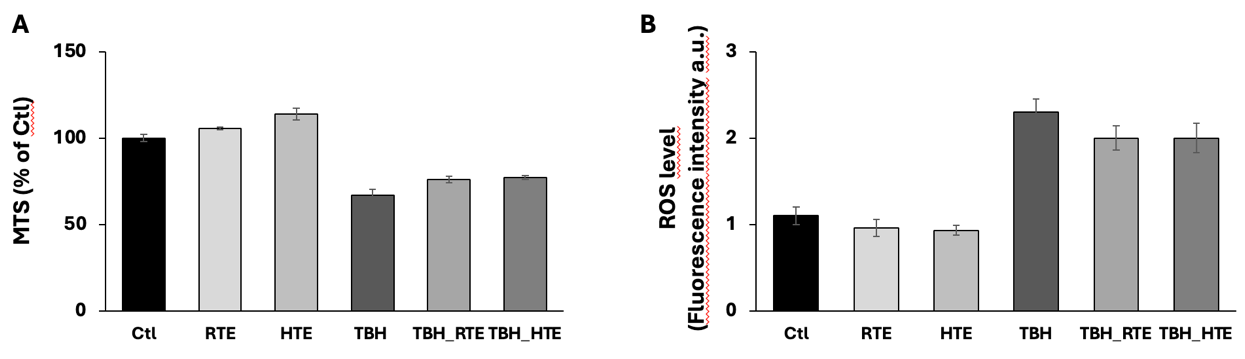
Figure S2**

Fig. S2 - Effects of RTE and HTE on cell viability and total ROS production in LAN5 cells challenged with tert-Butyl hydroperoxide (TBH). Cells were exposed overnight to RTE or HTE 2 µg/ml and then treated with TBH 0.125 mM for 1 h. **A)** Cell viability evaluated by MTS assay. **B)** Total ROS production assessed by DCFH-DA assay. The data are shown as means ± SD of three separate experiments at least

**Cell treatment**

*Cell culture*

The human neuroblastoma cell lines LAN5 (DSMZ Collection, ACC-673) were cultured as monolayer and grown in 100 mm diameter polystyrene dishes using Dulbecco**’**s Modified Eagle’s Medium (DMEM; Corning, New York, NY, USA) supplemented with 10% fetal bovine serum (FBS), 100 U/mL penicillin, and 100 μg/mL streptomycin (Corning). Cells were maintained in a humidified incubator at 37 °C with a 5% CO2 atmosphere.

*Cell viability Assays*

LAN5 cell viability was evaluated by MTS assay using the CellTiter 96 AQueous One Solution Cell Proliferation Assay Kit (Promega Corporation, Madison, WI, USA). LAN5 cells (6x104 cells/well in 100 µL growth medium) were seeded in a 96 -well plate and after 24 h were treated with RTE or HTE at different concentrations of polyphenols (0.125-16 µg/mL GAE) for 24 h. MTS assay was performed according to the manufacturer’s instructions. In brief, after the cell treatments, 20 µL of reagent solution were added into each well of untreated and treated cells. Plate was incubated 4 h at 37 °C in a humidified 5% CO2 atmosphere. Absorbance was read at 490 nm using the Multiplate reader iMark (BioRad, Hercules, CA, USA). Viability was expressed as percentage relative of untreated cells (control, Ctl).

*Assessment of total Reactive Oxygen Species (ROS) production in LAN5 cells*

Intracellular ROS level was evaluated by 2′,7′-Dichlorofluorescin diacetate (DCFH-DA) assay accordingly to Wang and Josef (Wang & Joseph, 1999) with few modifications. In brief, LAN5 cells were seeded in a 96-well black plate at a density of 6x104 cells/well in 100 µL growth medium for 24 h. Then, cells were untreated (control) or treated overnight with 2 µg/mL polyphenols of RTE or HTE. Following, after two gentle washes with PBS, serum free medium containing 100 µM of DCFH-DA weas added into each well and cells were incubated for 1 h at 37 °C in a humidified 5% CO2 atmosphere. DCFH-DA is a cell-permeable fluorogenic probe that, after crossing the cell membranes, is hydrolyzed by intracellular esterase to non-fluorescent DCFH. In the presence of ROS, DCFH is oxidized to fluorescent DCF giving origin to an intracellular green fluorescence. After the incubation at 37 °C, cells were washed three times with PBS and treated for 1 h with 0.125 mM of TBH to induce oxidative stress. The fluorescence resulting by production of intracellular ROS was measured in the Fluoroskan Ascent FL Thermo Scientific microplate reader (485 nm and 528 nm for excitation and emission wavelength, respectively). Results were expressed as intracellular ROS level by DCF fluorescence intensity. All treatments were performed in the dark.

The LAN5 data are expressed as the mean of at least three independent experiments ± Standard Deviations (SD). Results were compared using one-way analysis of variance with pairwise comparisons among treatments made using *t*-test. The analyses were performed using the Excel software. Results were considered statistically significant at **p<*0.05.
